# Apoptosis-related genes bridge inflammatory bowel disease and osteoporosis: A bioinformatic analysis

**DOI:** 10.1371/journal.pone.0356002

**Published:** 2026-08-12

**Authors:** Yue Su, Xiaohui Luo, Haitao Xu

**Affiliations:** 1 Anorectal Department, Wenshan Zhuang and Miao Autonomous Prefecture Hospital of Traditional Chinese Medicine, Wenshan Zhuang and Miao Autonomous Prefecture, Yunnan, China; 2 Department of Orthopedics, Shengjing Hospital of China Medical University, Shenyang, Liaoning, China; 3 Department of Orthopedics, The Affiliated Yongchuan Hospital of Chongqing Medical University, Chongqing, China; University of Vermont, UNITED STATES OF AMERICA

## Abstract

**Background:**

Inflammatory bowel disease (IBD) and osteoporosis (OP) often co-occur, with IBD accelerating OP onset, though the underlying mechanisms remain unclear.

**Methods:**

Based on gene expression data from the GEO database, differential expression analysis was performed using the limma package to identify genes dysregulated in both IBD and OP. Weighted Gene Co-expression Network Analysis (WGCNA) was employed to explore the associations between genes and phenotypes. Candidate genes were obtained by intersecting differentially expressed genes with key WGCNA modules, followed by Gene Ontology and Kyoto Encyclopedia of Genes and Genomes enrichment analyses. To further focus on apoptosis-related mechanisms, the candidate genes were intersected with an apoptosis-related gene set and refined using lasso regression. Core genes were identified through differential expression validation and receiver operating characteristic curve analysis. Subsequently, their potential functions and regulatory networks were explored via multidimensional analysis, gene set enrichment analysis, and Pearson correlation analysis.

**Results:**

WNT1 and S100A8 are closely associated with the progression of both inflammatory bowel disease and osteoporosis. Gene Set Enrichment Analysis revealed that WNT1 is primarily enriched in cell cycle checkpoint signaling, chromosome organization, neuroactive ligand signaling, and neuroactive ligand–receptor interaction. In contrast, S100A8 is mainly enriched in adaptive immune response, adaptive immune response based on somatic recombination of immune receptors built from immunoglobulin superfamily, inflammatory bowel disease, and leishmaniasis. Pearson correlation analysis showed that WNT1 is correlated with TSPAN32, POU3F3, NXPH3, EGFL7, LHX3, TNNI2, HMX1, FAM3A, IBTK, and ZNF747, while S100A8 is correlated with MMP3, SLC6A14, MMP10, S100A9, AQP9, S100A12, CXCL6, CXCR1, SLC38A4, and NCF2.

**Conclusions:**

WNT1 and S100A8, which are associated with apoptosis, play critical roles in both inflammatory bowel disease and osteoporosis, providing new potential therapeutic targets and a theoretical basis for clinical diagnosis and treatment.

## Background

Osteoporosis(OP) is a common systemic metabolic bone disease characterized primarily by reduced bone mass and deterioration of bone microarchitecture, which leads to decreased bone strength and a significantly increased risk of fragility fractures [1]. According to a previous report, the prevalence of OP among the elderly population in China reaches 33.49%, meaning that one in every three elderly individuals is affected [2]. With the accelerated aging of the global population, the number of OP patients is expected to rise further in the future [3]. This trend not only increases the medical burden but also severely impacts patients’ quality of life [4]. The most common complication of OP is osteoporotic fracture, with a lifetime fracture risk of up to 40% for affected patients. Such fractures mainly include vertebral compression fractures and hip fragility fractures, with hip fragility fractures accounting for over 40% of cases. Moreover, the probability of refracture after vertebroplasty for vertebral compression fractures is nearly 20%. Fractures can lead to loss of mobility and independence, further reducing patients’ quality of life. Additionally, due to prolonged bed rest during hospitalization, osteoporotic fracture patients face a significantly elevated risk of complications such as pneumonia or thromboembolic events, which are associated with an excess mortality rate of up to 20% within 12 months. This further exacerbates the public health burden [5–7]. Therefore, in-depth research into the mechanisms underlying the development and progression of OP is of great importance for the prevention and treatment of this disease.

Inflammatory bowel disease (IBD) is a chronic, heterogeneous inflammatory disorder of the intestines, primarily including Crohn’s disease and ulcerative colitis. Crohn’s disease can affect the entire gastrointestinal tract from the mouth to the anus, while ulcerative colitis lesions are mainly confined to the colon [8]. Its inflammatory features are chronic and relapsing, with clinical manifestations often including abdominal pain, weight loss, diarrhea, and other symptoms [9,10]. Persistent inflammation may lead to complications such as intestinal strictures, fistula formation, weight loss, malnutrition, and even malignancy [11]. According to incomplete statistics, IBD affects approximately 1.5 million Americans and 2.2 million Europeans [12,13], with a global prevalence estimated at 0.3% to 0.5% [14]. he etiology of IBD is complex, involving the interplay of multiple factors including genetics, environment, diet, immune responses, and neural regulation [15]. Due to its chronic and protracted course, patients often face increased treatment burdens, higher hospitalization rates, and significant impacts on their quality of life and social functioning [12,16].

In the treatment of IBD, glucocorticoids play a crucial role. For example, oral systemic glucocorticoids such as prednisone and prednisolone have been used for over 60 years to alleviate symptoms of IBD. Second-generation glucocorticoids (e.g., budesonide and beclomethasone dipropionate), due to their stronger anti-inflammatory activity, significant first-pass effect (resulting in low systemic bioavailability), and higher affinity for glucocorticoid receptors, have now become an important option for the treatment of IBD [17]. However, excessive use of glucocorticoids can inhibit the proliferation and differentiation of osteoblasts while promoting the apoptosis of osteoblasts and osteocytes, thereby inducing or exacerbating OP [18–22]. The prevalence of OP is significantly higher in patients with IBD [23]. The shared driving mechanisms behind the burden of these two diseases involve the impact of both IBD itself and its therapeutic agents on bone metabolism. Therefore, IBD is one of the significant etiological factors for osteoporosis, and elucidating the common pathogenesis and related molecular targets linking IBD and OP is of great importance for clinical prevention and treatment.

The aim of this study is to identify key genes commonly associated with both IBD and OP by integrating bioinformatics analytical approaches, thereby uncovering potential shared genetic mechanisms between the two diseases. This research seeks to provide a theoretical foundation for mitigating bone loss related to IBD and to offer novel potential targets for the prevention and treatment of OP in patients with IBD.

## Methods

### Sample source

The data related to IBD and OP were both obtained from the GEO database. The expression matrix for IBD was derived from the GSE126124 dataset, from which tissue-related expression profiles were selected, including 21 normal tissues and 57 diseased tissues, with the platform type GPL6244. The expression matrix for OP was sourced from the GSE56815 dataset, which included 40 normal samples and 40 OP samples, using the platform GPL96. The list of apoptosis-related genes was acquired from the GeneCards website (https://www.genecards.org/), with data collection up to December 1, 2025. To identify genes more closely associated with the apoptosis process, genes with a relevance score ≥ 5 were selected for subsequent analysis.

### Differential analysis

To analyze the differentially expressed genes in the GSE126124 and GSE56815 datasets, this study utilized the limma package in R for differential expression analysis. To ensure statistical significance of the identified genes, the screening threshold for differentially expressed genes was set at a p < 0.05. The resulting differentially expressed genes will be used for subsequent analyses.

### WGCNA analysis

Weighted Gene Co-expression Network Analysis (WGCNA) was performed on the GSE126124 dataset using the WGCNA package to investigate the association between gene modules and disease status. The analysis parameters were set as follows: power = 8, TOMType = “signed”, minModuleSize = 30, and mergeCutHeight = 0.20. All genes included in the analysis had a standard deviation greater than zero, ensuring the exclusion of potential outliers in advance. The study employed the “pickSoftThreshold” function in WGCNA to determine the optimal soft threshold (b = 8) for constructing an unscaled network, based on which distinct gene modules were identified. Modules with similar expression patterns were merged using a threshold of 0.20, while a minimum of 30 modules were retained. The resulting modules comprised gene sets with similar co-expression characteristics.

### Identification of key genes

To identify key genes involved in both IBD and OP, we performed an integrated analysis of multi-source gene expression data. First, the differentially expressed genes (DEGs) from the GSE126124 dataset were intersected with genes from the IBD-related WGCNA modules to obtain a set of candidate genes. Subsequently, this gene set was further intersected with DEGs from the GSE56815 OP dataset to screen for potential key genes that may be jointly involved in both disease processes. To further focus on apoptosis-related mechanisms, the intersected genes were then cross-screened with an apoptosis-related gene set. The final set of genes identified was used for subsequent in-depth analysis. The results of all intersection analyses were visualized using Venn diagrams generated via the Sangerbox platform (http://www.sangerbox.com/tool) [24].

### Enrichment analysis

Functional enrichment analysis for the intersecting genes of IBD and OP was performed using the Metascape platform (https://metascape.org/gp/index.html#/main/step1). This analysis included Gene Ontology (GO) enrichment and Kyoto Encyclopedia of Genes and Genomes (KEGG) pathway enrichment to identify key biological processes, molecular functions, cellular component, and associated signaling pathways. The enriched terms were clustered based on functional similarity, from which the most significantly enriched and representative terms were selected. A statistical significance threshold of p < 0.05 was applied in the analysis.

### Integrating data and analyzing related genes

We integrated expression profiles of GSE126124 and GSE56815, and corrected source-derived batch heterogeneity with the ComBat algorithm embedded in the sva package to avoid biases limiting the generalizability of our findings. Subsequently, the hmisc package was employed to analyze genes highly correlated with key gene expression. Based on the results of pearson correlation analysis, corresponding scatter plots were generated using the ggpubr package to visually demonstrate the expression associations between genes.

### Machine learning screening

The lasso regression machine learning method was employed, using the glmnet package, to perform gene screening on the GSE126124 and GSE56815 datasets. By introducing a regularization penalty term, lasso regression optimizes variable selection, effectively identifying key genes while reducing the risk of overfitting and minimizing interference from irrelevant genes. Prior to analysis, the data were standardized, and cross-validation was used to determine the optimal regularization parameter (lambda). Genes with non-zero coefficients were retained as significantly associated genes for subsequent research. To validate their discriminative efficacy, receiver operating characteristic (ROC) analysis was further conducted. By plotting sensitivity versus specificity curves, the predictive accuracy of the selected genes in distinguishing disease from normal states was evaluated. The area under the curve (AUC) was used to quantify the classification ability of each gene, with higher AUC values indicating better performance. Finally, single-gene gene set enrichment analysis (GSEA) was performed on the key genes to explore their functional relevance. By identifying significantly enriched pathways in the dataset, GSEA helps reveal the biological processes in which each gene may be involved.

## Result

### Analyze differentially expressed genes and screen for key genes

**Fig 1:**
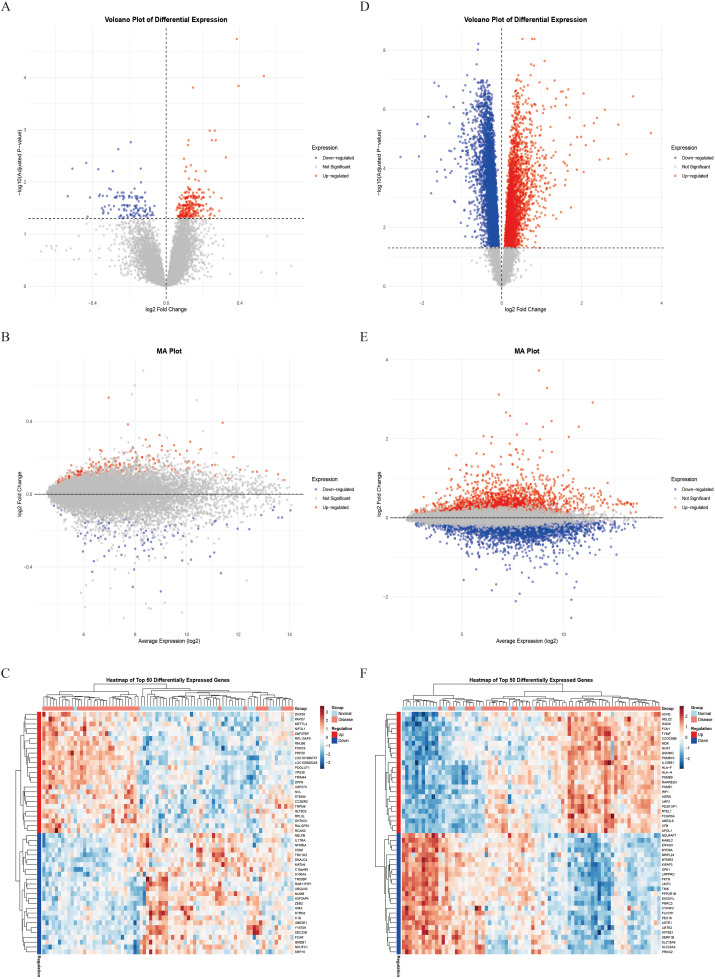
Differential gene analysis results for osteoporosis and inflammatory bowel disease. A: Volcano plot of differentially expressed genes in osteoporosis; B: MA plot of differentially expressed genes in osteoporosis; C: Heatmap of differentially expressed genes in osteoporosis; D: Volcano plot of differentially expressed genes in inflammatory bowel disease; E: MA plot of differentially expressed genes in inflammatory bowel disease; F: Heatmap of differentially expressed genes in inflammatory bowel disease.

First, to identify genes closely associated with IBD and OP, we retrieved the GSE56815 and GSE126124 datasets from the GEO database and performed differential expression analysis. In the GSE56815 dataset, we identified OP-related differentially expressed genes, screening a total of 378 genes with a p-value < 0.05, among which 263 genes were upregulated and 115 genes were downregulated in the OP group. The results were visualized using volcano plots, MA plots, and heatmaps (Fig 1A–1C). Subsequently, in the GSE126124 dataset, we analyzed IBD-related differentially expressed genes, identifying a total of 9,039 genes with a p-value < 0.05, including 3,889 upregulated and 5,150 downregulated genes. The corresponding results were presented as volcano plots, heatmaps, and MA plots (Fig 1D–1F).

**Fig 2:**
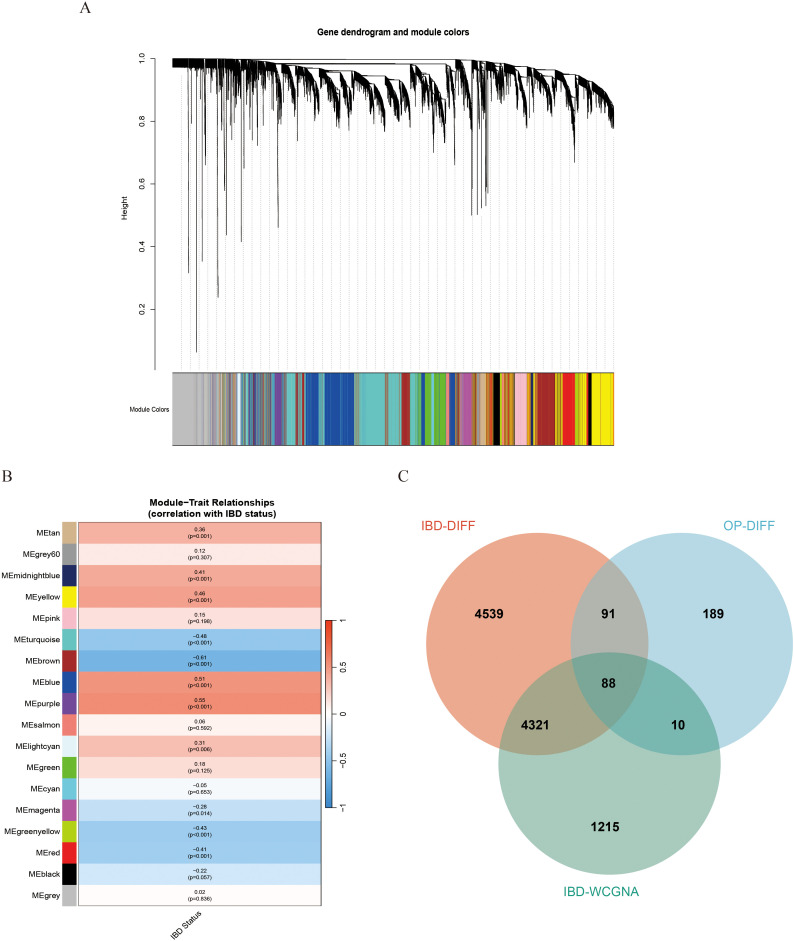
WGCNA analysis results of inflammatory bowel disease and identification of key genes. A: Threshold visualization of WGCNA analysis results; B: Display of different modules from the WGCNA analysis; C: Identification of key genes shared between inflammatory bowel disease and osteoporosis.

To further screen for potential key genes that may play important functional roles and are closely associated with IBD, we performed WGCNA analysis on the GSE126124 dataset. Through this analysis, 5,634 module genes related to IBD were identified (p < 0.05), with the corresponding results shown in Fig 2A and 2B. Subsequently, to screen for genes simultaneously associated with both IBD and OP, a multi-step gene intersection analysis was conducted. The specific steps were as follows: first, the differentially expressed genes from the GSE126124 dataset were intersected with the module genes identified by WGCNA, resulting in 4,417 genes closely related to IBD. Then, these genes were further intersected with the 378 OP-related differentially expressed genes screened from the GSE56815 dataset. Ultimately, 88 co-expressed genes were obtained for subsequent in-depth analysis (Fig 2C).

### Gene enrichment analysis

**Fig 3:**
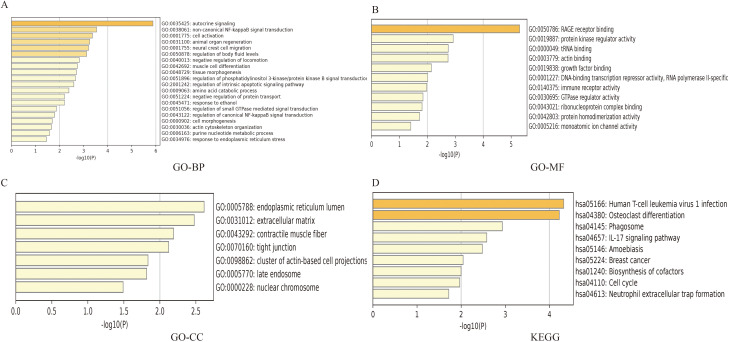
KEGG and GO enrichment analysis of key genes. A: GO-BP enrichment analysis results of key genes; B: GO-MF enrichment analysis results of key genes; C: GO-CC enrichment analysis results of key genes; D: KEGG enrichment analysis results of key genes.

We further performed GO and KEGG enrichment analyses on these 88 genes. The GO enrichment results indicated that these genes are significantly associated with pathways related to the non-canonical NF-κB signal transduction, cell activation, RAGE receptor binding, and protein kinase regulatory activity (Fig 3A and 3B). The KEGG enrichment analysis revealed that these genes are primarily enriched in the osteoclast differentiation and IL-17 signaling pathways (Fig 3C and 3D).

### Machine learning

**Fig 4:**
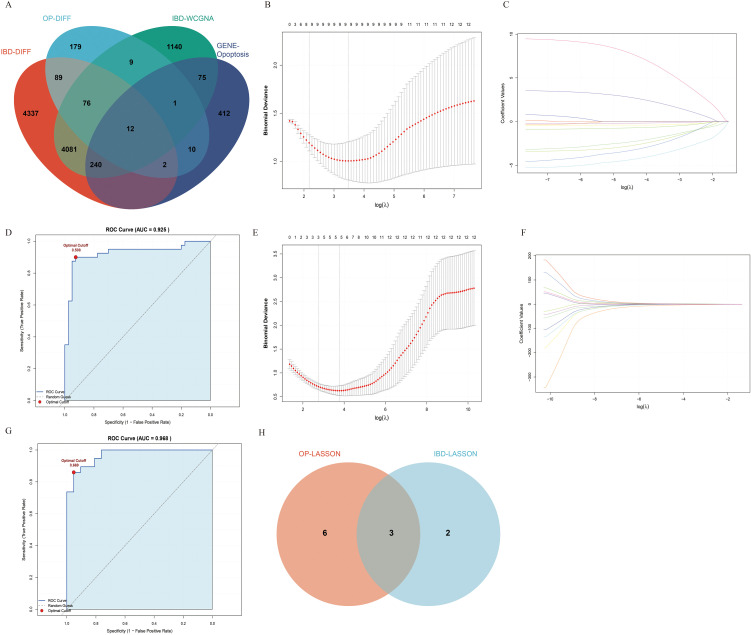
Identification of apoptosis-related genes and machine learning screening. A: Determination of apoptosis-related genes; B-D: Key genes screened by lasso method based on the osteoporosis dataset; E-G: Key genes screened by lasso method based on the inflammatory bowel disease dataset; H: Intersection of key genes screened by lasso method from both datasets.

Apoptosis plays a critical role in the pathological processes of both OP and IBD [25,26]. Therefore, we performed an intersection analysis between the previously identified shared differentially expressed genes and a set of apoptosis-related genes, identifying a total of 12 genes: NFKBIA, CCND1, HSPB1, SOCS3, NFKB2, SLC25A5, S100A9, S100A4, MITF, S100A8, WNT1, and HNRNPA1 (Fig 4A). To further investigate the roles of these genes in both diseases, we conducted lasso regression analyses on the OP and IBD datasets, respectively. First, in the analysis of the OP dataset, lasso regression results showed that 9 out of the 12 genes were closely associated with OP, namely NFKBIA, HSPB1, SOCS3, NFKB2, SLC25A5, S100A4, MITF, S100A8, and WNT1 (Fig 4B–4D). Subsequently, in the analysis of the IBD dataset, lasso regression identified 5 genes associated with IBD, including HNRNPA1, MITF, S100A8, WNT1, and CCND1 (Fig 4E–4G). By intersecting the results from the two lasso regression analyses, we ultimately identified 3 genes simultaneously associated with both IBD and OP: MITF, S100A8, and WNT1 (Fig 4H).

**Fig 5:**
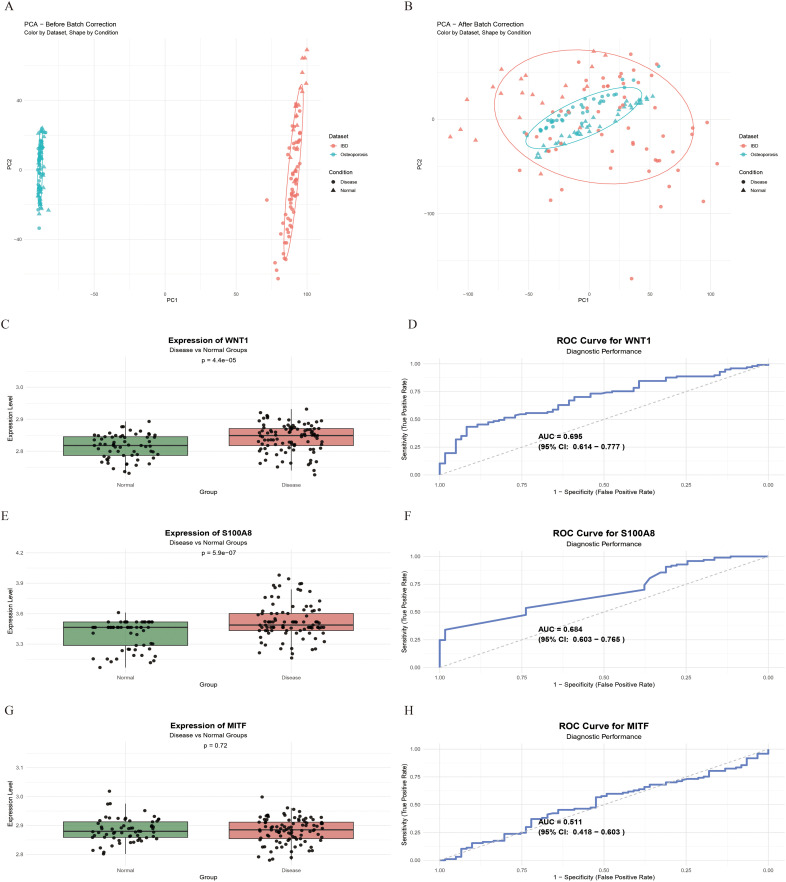
Batch correction results and box plots with ROC curves for key genes. A: PCA plot of merged data before batch correction; B: PCA plot of merged data after batch correction; C: Box plot showing differential expression results of WNT1 between disease and normal groups; D: ROC curve for WNT1; E: Box plot showing differential expression results of S100A8 between disease and normal groups; F: ROC curve for S100A8; G: Box plot showing differential expression results of MITF between disease and normal groups; H: ROC curve for MITF.

To further evaluate the diagnostic value of the three genes—S100A8, WNT1, and MITF—in IBD and OP, we merged the two datasets and performed batch effect correction on the combined data to eliminate systematic bias potentially introduced by non-biological factors (Fig 5A and 5B). In the corrected data, we conducted differential expression analysis and receiver operating characteristic (ROC) performance assessment for each of these three genes. The results showed that S100A8 and WNT1 were significantly upregulated in the disease groups, with area under the curve (AUC) values of 0.695 and 0.684, respectively, indicating strong diagnostic discriminative ability for disease status (Fig 5C–5F). In contrast, MITF showed no significant differential expression between disease and control groups, with an AUC value of only 0.511 (close to 0.5) and a 95% confidence interval that included 0.5, suggesting that this gene did not demonstrate significant diagnostic discriminative efficacy in the current study (Fig 5G and 5H). Therefore, we ultimately identified S100A8 and WNT1 as key genes closely associated with both IBD and OP.

### Enrichment analysis of WNT1 and S100A8

**Fig 6:**
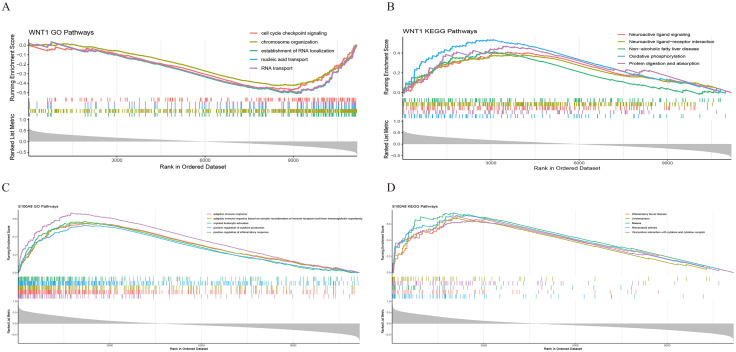
GO and KEGG enrichment analysis results of key genes. A: GO enrichment analysis results of WNT1; B: KEGG enrichment analysis results of WNT1; C: GO enrichment analysis results of S100A8; D: KEGG enrichment analysis results of S100A8.

To further investigate the potential pathways involving the two genes, S100A8 and WNT1, we performed Gene Set Enrichment Analysis (GSEA) on them. The GO enrichment results for WNT1 showed that it was primarily and significantly enriched in pathways related to cell cycle checkpoint signaling and chromosome organization. KEGG enrichment analysis indicated that WNT1 was significantly enriched in neuroactive ligand signaling and neuroactive ligand-receptor interaction, while its enrichment distribution was also associated with oxidative phosphorylation. For the S100A8 gene, GO analysis results demonstrated that it was mainly and significantly enriched in pathways involving adaptive immune response and adaptive immune response based on somatic recombination of immune receptors built from immunoglobulin superfamily. KEGG analysis suggested that S100A8 was significantly enriched in pathways related to inflammatory bowel disease and leishmaniasis (Fig 6A–6D)

### Genes potentially associated with S100A8 and WNT1

**Fig 7:**
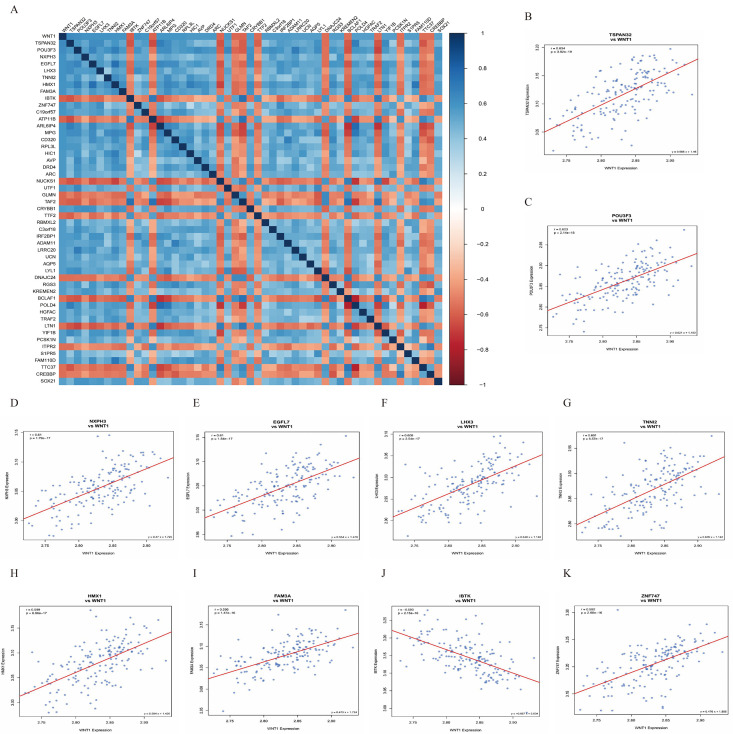
Analysis results of genes closely related to WNT1 expression. A: Heatmap of the 50 genes most correlated with WNT1 expression; B-K: Scatter plots of the 10 genes most correlated with WNT1 expression.

**Fig 8:**
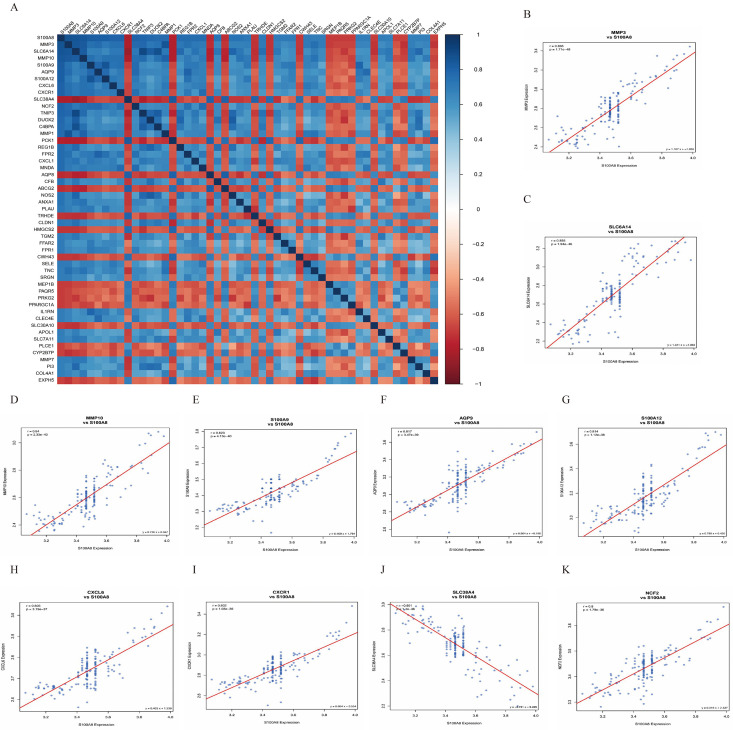
Analysis results of genes closely related to S100A8 expression. A: Heatmap of the 50 genes most correlated with S100A8 expression; B-K: Scatter plots of the 10 genes most correlated with WNT1 expression.

Furthermore, through correlation analysis on the integrated data, we identified the genes most closely associated with the expression of WNT1 and S100A8 and listed the top ten genes with the strongest correlations. The ten genes potentially highly correlated with WNT1 expression are TSPAN32, POU3F3, NXPH3, EGFL7, LHX3, TNNI2, HMX1, FAM3A, IBTK, and ZNF747 (Fig 7A–7K). The ten genes potentially highly correlated with S100A8 expression are MMP3, SLC6A14, MMP10, S100A9, AQP9, S100A12, CXCL6, CXCR1, SLC38A4, and NCF2 (Fig 8A–8K). These genes may be closely related to WNT1 and S100A8 in terms of function or regulation.

## Discussion

To date, the treatment of osteoporosis primarily relies on pharmacological interventions and educational efforts targeting patients’ lifestyles. With the intensifying trend of global population aging, enhancing public awareness of OP-related risks has become particularly important. Extensive research confirms a significant association between IBD and OP. Patients with IBD generally exhibit reduced bone mineral density, and as the disease progresses, the incidence of OP further increase [27]. These patients often experience progressive systemic inflammation, deficiencies in nutrients (such as vitamin D), hypogonadism, and may undergo glucocorticoid therapy or intestinal resection. Collectively, these factors exacerbate the risk of OP in patients with IBD [28].

In this study, based on the GSE126124 and GSE56815 datasets from the GEO database, we employed comprehensive bioinformatics methods to analyze the expression profiles of IBD and OP. Differential expression analysis revealed 378 differentially expressed genes in the OP dataset. In the IBD dataset, WGCNA analysis identified 5,634 genes in disease-related modules, while differential expression analysis screened 9,039 differentially expressed genes. Through intersection analysis of the differentially expressed genes from both IBD and OP, 88 key genes potentially involved in the pathogenesis of both diseases were identified. Further KEGG and GO enrichment analyses showed that these genes were primarily enriched in the non-canonical NF-κB signaling pathway, osteoclast differentiation, and the IL-17 signaling pathway. These pathways play significant roles in the pathological mechanisms of both IBD and OP, further suggesting the synergistic regulatory effects of these genes in the two diseases.

Further machine learning analysis using lasso regression was applied to these genes, and through intersection screening, MITF, S100A8, and WNT1 were ultimately identified as playing key roles in both OP and IBD. Subsequently, differential expression analysis and receiver operating characteristic (ROC) curve evaluation of these three genes confirmed that WNT1 and S100A8 exhibit significant diagnostic and functional relevance in both diseases. Previous studies have highlighted the critical roles of WNT1 in both conditions. Ortiz-Masiá et al. found that under hypoxic conditions, increased WNT1 expression in macrophages activates the mTOR pathway, inhibits autophagy, and impairs epithelial cell autophagic function, suggesting WNT1 may contribute to the development and progression of IBD [29]. In OP, WNT1 plays a particularly important role under mechanical stimulation by activating β-catenin to regulate Plat expression and induce the expression of Runx2 and Sp7, thereby promoting bone formation and alleviating OP [30]. Meanwhile, S100A8 has been confirmed as a sensitive inflammatory biomarker in IBD [31], with significantly elevated mRNA expression levels in patients, especially during active disease phases [32]. In OP, Wang et al. demonstrated that S100A8/A9 secreted during the whitening of brown adipose tissue can inhibit osteoblast differentiation [33]. In summary, WNT1 plays an important role in both the regulation of autophagy in intestinal epithelial cells and osteogenic differentiation in OP, while S100A8 is not only closely associated with the severity of inflammation in IBD but also involved in regulating osteoblast differentiation. The findings of this study suggest that WNT1 and S100A8 may serve as key molecular links between IBD and OP, holding promise as potential therapeutic targets for both diseases with significant translational medical value.

In this study, by integrating transcriptomic data and bioinformatics analysis, we further elucidated the association between IBD and OP and explored potential common mechanisms and key molecular sites between the two diseases. The results not only validated previously reported pathological pathways related to both diseases, such as the non-canonical NF-κB signaling pathway, osteoclast differentiation, and the IL-17 signaling pathway, but also identified WNT1 and S100A8 as potential key targets linking the two diseases through machine learning and ROC analysis. These targets hold promise for providing new therapeutic directions for the co-management of IBD and OP, contributing to a deeper understanding of their intrinsic molecular regulatory networks and offering a theoretical basis for developing more effective treatment strategies.

Nevertheless, the present study still has several limitations. This study primarily relied on bioinformatics mining of public databases and lacked further validation through molecular biology experiments and clinical samples. Therefore, future research should involve in vitro and in vivo experiments, as well as clinical cohort analyses, to further validate and expand upon the findings of this study.

## Conclusion

The WNT1 and S100A8 genes may play crucial roles in the development and progression of IBD and OP, providing potential therapeutic targets for both conditions and contributing to the advancement of novel drug development for patients with these diseases. However, the current findings still require experimental validation. Future work should focus on verifying and elucidating the specific functional mechanisms of these genes through experimental approaches. A deeper understanding of the shared regulatory mechanisms underlying both diseases holds promise for developing new clinical strategies for the treatment of IBD and OP, ultimately enabling more precise and effective disease management.

## Abbreviations

OP: Osteoporosis
IBD: Inflammatory bowel disease
WGCNA: Weighted Gene Co-expression Network Analysis
DEGs: differentially expressed genes
GO: Gene Ontology
KEGG: Kyoto Encyclopedia of Genes and Genomes
ROC: receiver operating characteristic
AUC: area under the curve
