# Correction: Apoptosis-related genes bridge inflammatory bowel disease and osteoporosis: A bioinformatic analysis

**DOI:** 10.1371/journal.pone.0359291

**Published:** 2026-09-25

**Authors:** Yue Su, Xiaohui Luo, Haitao Xu

There are errors in the author affiliations. The correct affiliations are as follows:

Yue Su^2^, Xiaohui Luo^3^, Haitao Xu¹^,^*

**1** Department of Orthopedics, The Affiliated Yongchuan Hospital of Chongqing Medical University, China, **2** Anorectal Department, Wenshan Zhuang and Miao Autonomous Prefecture Hospital of Traditional Chinese Medicine, China, **3** Department of Orthopedics, Shengjing Hospital of China Medical University, China
